# Modulation of innate lymphoid cells by enteric bacterial pathogens

**DOI:** 10.3389/fimmu.2023.1219072

**Published:** 2023-07-06

**Authors:** Prakash Sah, Lauren A. Zenewicz

**Affiliations:** Department of Microbiology and Immunology, College of Medicine, The University of Oklahoma Health Sciences Center, Oklahoma City, OK, United States

**Keywords:** GI tract, bacteria, IL-22, ILCs, innate lymphoid cells, modulation of immune response

## Abstract

Innate lymphoid cells (ILCs) are key regulators of tissue homeostasis, inflammation, and immunity to infections. ILCs rapidly respond to environmental cues such as cytokines, microbiota and invading pathogens which regulate their function and phenotype. Even though ILCs are rare cells, they are enriched at barrier surfaces such as the gastrointestinal (GI) tract, and they are often critical to the host’s immune response to eliminate pathogens. On the other side of host-pathogen interactions, pathogenic bacteria also have the means to modulate these immune responses. Manipulation or evasion of the immune cells is often to the pathogen’s benefit and/or to the detriment of competing microbiota. In some instances, specific bacterial virulence factors or toxins have been implicated in how the pathogen modulates immunity. In this review, we discuss the recent progress made towards understanding the role of non-cytotoxic ILCs during enteric bacterial infections, how these pathogens can modulate the immune response, and the implications these have on developing new therapies to combat infection.

## Introduction

Innate lymphoid cells (ILCs) are rare lymphocytes that are primarily tissue-resident and often present in mucosal tissues. Lacking specific antigen receptors, ILCs are mainly activated by cytokines and rapidly respond to infections. ILCs represent innate counterparts of T cells as they share several phenotypic and functional features (1). ILCs can be classified into four distinct subsets: NK cells, ILC1s, ILC2s, and ILC3s based on expression of lineage-specifying transcription factors (TF), surface markers and effector functions (2, 3). We will focus on ILCs as NK cells have been well-recognized and studied for decades. ILC1s express T-bet and often protect against intracellular pathogens via production of IFNγ but generally are non-cytotoxic (4). ILC2s express the lineage defining TF GATA3 and produce the type 2 cytokines IL-5 and IL-13, and lesser amounts of IL-4 and IL-9. ILC2s are often key to mediating immune responses to helminths and allergens (5). ILC3 development depends on the TF RORγt and these cells produce IL-22 to maintain tissue barrier function and protect against extracellular pathogens (5, 6). Further, ILCs can have cellular plasticity and can transdifferentiate to other ILC subtypes depending upon the cytokine and/or environmental milieu (3–5).

Mucosal surfaces such as those of the GI tract harbor billions of microorganisms as part of the normal microbiota, which is the first line of defense against bacterial pathogens. Nevertheless, many pathogens enter the host through mucosal surfaces causing infection. The immune system has evolved to provide resistance to pathogens while maintaining homeostasis with the microbiota. ILCs are enriched in mucosal surfaces and orchestrate the early defense against invading pathogens. Although ILC3s are cast to be the ILCs that respond to extracellular bacterial infection, ILC1s and ILC2s also can contribute to the immune responses against these pathogens. For every well-honed immune response to combat a pathogen, a host may have a less than perfect response because of many possible reasons, with a primary one being that the pathogen can fight back. Enteric bacteria produce many toxins and other effector molecules that target host cells and can cause apoptosis or interfere in signaling pathways.

**Figure 1:**
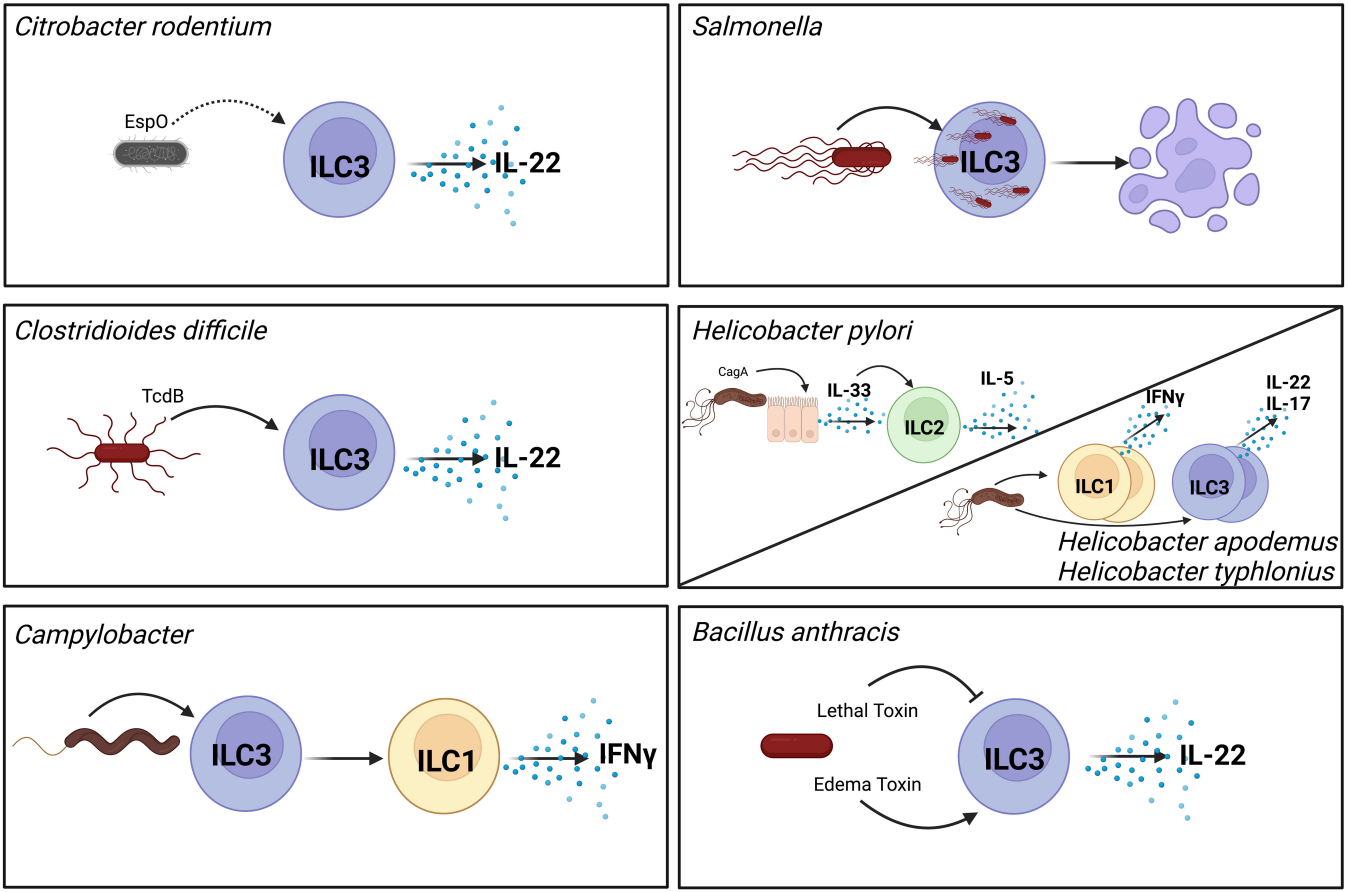
Pathogenic bacteria modulate ILC function through different mechanisms. *Citrobacter rodentium* produces the effect EspO which indirectly contributes to activation of ILC3s and their production of secreted IL-22 (7). As an intracellular pathogen *Salmonella* invades ILC3s which induces their pyroptosis and reduces their numbers in the GI tract (8). Toxin B (TcdB) produced by *Clostridioides difficile* directly activates ILC3s to produce IL-22 (9). *Helicobacter pylori* activates epithelial cells to produce IL-33 in a CagA-dependent manner. This IL-33 in turns activates ILC2s in the stomach to produce IL-5 (10, 11). Non-gastric *Helicobacter* species increase frequencies of cytokine producing ILC1s and ILC3s (12). *Campylobacter* infection induces conversion of ILC3s to ILC1s that can produce IFNγ (13). *Bacillus anthracis* secretes two toxins with opposing effects on ILC3s. Lethal toxin inhibits MAPK signaling which inhibits ILC3 activation (14). In contrast, edema toxin directly activates ILC3s to produce IL-22 (15). Direct interactions are shown with solid arrows, indirect interactions are shown with dashed arrows. Created with BioRender.com.

Recent studies have highlighted roles of ILCs during infection and modulation of ILC functions by pathogenic bacteria. Although challenging to test the precise role of virulence factors on ILCs in *in vivo* models due to the requirement for virulence factors for infectivity and the limitations on the tools to specifically target ILCs and not other immune cells, we have discerned much on how ILCs respond to different gastrointestinal (GI) bacterial infections and how the pathogen may affect this response (**Figure 1**). Here, we present the current state of the field on modulation of non-cytotoxic ILC functions by enteric bacterial pathogens and how it contributes to pathogenesis or protection against these pathogens.

## Modulation of ILC function by bacterial pathogens

### Citrobacter rodentium

*C. rodentium* is a murine Gram-negative pathogen that is widely used to model the human pathogen enteropathogenic *Escherichia coli* (EPEC) (16). Like EPEC, *C. rodentium* secretes effectors via a type 3 secretion system (T3SS) into intestinal epithelial cells to modulate host cell processes and establish infection (17). The utility of the *C. rodentium* infection model extends beyond bacterial pathogenesis studies to reveal fundamental aspects of mucosal immunity (18). *C. rodentium* infection elicits both innate and adaptive immune responses that are important for control and clearance of the pathogen in the initial and late phases of infection, respectively. ILC3s are critical immune cells in early control of *C. rodentium* (19–22). Following infection, macrophages and dendritic cells (DCs) are activated by bacterial pathogen-associated molecular patterns (PAMPs) and secrete inflammatory cytokines such as IL-23 and IL-1β which activate ILC3s (23–27). Upon activation, ILC3s rapidly produce IL-22, a critical cytokine in control of *C. rodentium* infection (5, 19, 28). ILC3s represent a major early source of IL-22 in infection (21, 29), where the cytokine upregulates antimicrobial peptides and mucin production promoting intestinal barrier resistance (19, 28).

*C. rodentium* T3SS effectors modulate innate immune responses (18). It is not known whether *C. rodentium* directly interacts with ILC3s. However, a recent study showed that the T3SS effector EspO modulates IL-22 signaling (7). While the study reported infection with *C. rodentium* lacking EspO reduced IL-22 secretion by colonic explants and a subsequent reduction in antimicrobial peptides, no change in the frequency of IL-22 producing ILC3s or T cells was observed (7). It is not clear why a *C. rodentium* effector would upregulate IL-22. Modulation of IL-22 by other pathogens is known to promote colonization (30) but this is unlikely in the case of *C. rodentium* as IL-22 is well-described to be protective in *C. rodentium* infection (19). *C. rodentium* is also known to modulate metabolism of intestinal epithelial cells (IECs) (18). T3SS effectors, Map and EspF, cause mitochondrial disruption leading to shift in IEC metabolism to aerobic glycolysis causing increased oxygenation of the intestinal mucosa that supports *C. rodentium* colonization (31–33). Given the role of hypoxia in regulating ILC3 function (34), the question arises whether this increased oxygenation of the GI tract caused by *C. rodentium* T3SS effectors modulate ILC3. Lastly, *C. rodentium* infection can cause metabolic rewiring of ILC3s, enhancing their proliferation and cytokine production (35). These “trained ILC3s” show properties of “innate memory” and are better in controlling re-infection than naive ILC3s in mice (35). In summary, *C. rodentium* infection has been a valuable model to learn much about pathogen-ILC interactions.

### Salmonella

*Salmonella enterica* encompasses several serovars that cause GI infection (36, 37). In developing countries, typhoidal serovars, S. Typhi and S. Paratyphi, cause enteric fever. Non-typhoidal serovars such as *S. enterica* serovar Typhimurium (S. Typhimurium) cause food-borne gastroenteritis that are usually self-limiting but can lead to disseminated infection (36). S. Typhimurium is the most widely studied serovar and findings reviewed here pertain to this serovar. Like many other Gram-negative GI pathogens, S. Typhimurium encodes for T3SS, which in *Salmonella* is two separate T3SS systems that secrete an arsenal of virulence factors (37).

Interferon-γ (IFNγ) is a key cytokine in defense against *Salmonella* infections (38–41). The main innate source of IFNγ during *Salmonella* GI infection is NKp46+ T-bet+ ILCs that have decreased RORγt levels (41). The IFNγ production by these ILCs during infection is driven by IL-12 while IL-23 has no significant effect (41). Although ILCs are mainly tissue-resident cells, Kastele et al. showed that *Salmonella* infection increased the migratory RORγt+ T-bet+ ILC population in the mesenteric lymph nodes and contributed to IFNγ production (42). Further refinement in identifying the IFNγ-producing cells, has shown significant IFNγ production by ILC1s but not ILC3s (8). IFNγ regulates mucin production during *Salmonella* infection and deficiency of the mucin MUC2 increases susceptibility to infection in a mouse model (39, 43). In line with this, depletion of ILCs or genetic ablation of IFNγ production by ILCs, results in impaired mucus production (41). Additionally, IFNγ-producing ILCs also contribute to intestinal inflammation as interference with IFNγ production by ILCs leads to reduced inflammation (41). These studies suggest that IFNγ production by ILCs induced by *Salmonella* infection can be both protective as well as pathologic.

IL-22 is upregulated during *Salmonella* infection (44). ILC3s via production of IL-22 and lymphotoxin-α mediate *in vitro* fucosylation of IECs (45). *In vivo* ILC3s mediate fucosylation during *Salmonella* infection and mice deficient in intestinal fucosylation are more susceptible to infection (45). Subsequent studies have reported that IL-22 is not protective in *Salmonella* infection. In fact, increased IL-22 promotes *Salmonella* infection by improving its ability to compete with microbiota (30, 46). This is attributed to its resistance to the antimicrobial peptides induced by IL-22 (30, 47, 48). In line with these observations, a recent study found that *Salmonella* induced IL-22 in ILC3s during infection which promoted infection (8). Flagellin activates antigen-presenting cells to produce IL-23, which in turn activated ILC3s. Further, *Salmonella* directly invade ILC3s leading to capase-1-mediated pyroptosis of ILC3s in a flagellin-independent manner. ILC3 depletion leads to less mortality and reduced disease severity in *Salmonella* infected mice (8). Thus, *Salmonella*-induced ILC3 pyroptosis is a possible host defense mechanism against *Salmonella* by limiting innate IL-22 production after early induction which benefits the bacteria (8). However, the exact signal(s) that induces ILC3 pyroptosis remain unknown. This example of bacterial modulation of ILC3s by regulating cell death is a bacterial defense mechanism that may prove to be relevant to other GI pathogens.

### Clostridioides difficile

*C. difficile* colitis is the most common nosocomial GI infection occurring in patients with perturbed microbiota owing to use of broad-spectrum antibiotics (49). *C. difficile* strains can encode for three different toxins: toxin A (TcdA), toxin B (TcdB) and *C. difficile* transferase (CDT) (50). The combined action of these toxins results in disruption of host cytoskeleton in the GI epithelium and ultimately loss of epithelial integrity. Toxin-mediated inflammation can result in symptoms ranging from mild diarrhea to pseudomembranous colitis (50).

Several studies have described the roles of ILCs during *C. difficile* infection (CDI) (51–53). *Nfil3* ^-/-^ mice, which due to a lack of the transcriptional regulator nuclear factor, interleukin 3 regulated (NFIL3) have reduced numbers of ILCs owing to a developmental defect in ILC maturation, are highly susceptible to CDI (54). Further, *Rag2^-/-^ γ_c_* ^-/-^ mice lacking both adaptive immune cells and ILCs have increased mortality following CDI compared to RAG-deficient, lacking only adaptative immunity cells or wild-type mice (51). IFNγ is increased in *Rag1^-/-^* mice following CDI and selective loss of ILC1s or ILC1-derived IFNγ leads to increased disease severity and mortality (51). The same study also reported an increase in ILC3-associated cytokines such as IL-22 and IL-17 following CDI. However, selective loss of ILC3s or the ILC3-associated cytokine IL-22 exhibited only a modest effect on CDI recovery (51), at least in the absence of an adaptive immune response. In contrast to this, Hasegawa et al. reported increased mortality following CDI in IL-22-deficient mice. Although, lack of IL-22 did not alter *C. difficile* burden and intestinal damage, IL-22 was important in clearance of pathobionts that translocated following intestinal damage by regulating the complement system (55). Together, these studies show that ILC3s and IL-22 are likely more important during the later stage of infection by preventing translocation of pathobionts whereas ILC1s are more important in the early defense against CDI. The protective role of ILC3s and/or IL-22 in context of CDI has been further confirmed by other studies (34, 53, 56, 57).

ILC2s are important players in immunity against helminth infection and allergic response in the GI tract (5). However, these cells have been recently shown to be important in bacterial infections, including *C. difficile* (52). IL-33, an activator of ILC2s, was found to be upregulated during CDI in cecal tissues of infected mice. IL-33 protected against CDI by activating ILC2s (52). IL-33-activated ILC2s increased mucin production by goblet cells, improving epithelial barrier function as well as increasing the number of eosinophils in the colon that are protective during CDI (52, 58, 59).

Although, the roles of different ILC subsets have been studied in context of CDI, whether *C. difficile* or its toxins directly interact with ILCs remains poorly defined. Recently, we reported that TcdB directly activates ILC3s *in vitro*, inducing IL-22 and other effector molecules (9). The TcdB-mediated activation of ILC3s required the toxin’s enzymatic activity and was in part mediated by inactivation of the small GTPase CDC42 (9). Gene expression analysis revealed that the toxin-mediated activation of ILC3s is distinct from IL-1β-mediated activation (9). Given the protective effects of IL-22 during *C. difficile* infection, the toxin mediated activation of ILC3s seems surprising. IL-22 may shape the microbiota to favor *C. difficile* over other species that the pathogen competes for resources. *In vivo* validation of this observation and function of toxin activated-ILC3s remains to determined.

### *Helicobacter* Spp.

*Helicobacter pylori* is a highly prevalent Gram-negative pathogen that infects half of the world’s population (60). It causes gastritis and is the strongest risk factor for gastric adenocarcinoma (61). The major virulence factors of *H. pylori* are type four secretion system (T4SS) effectors, which includes cytotoxin-associated gene A product (CagA) (61). ILC2s are the predominant ILC population in the stomach and increase in number during *H. pylori* infection (62, 63). IL-33 is increased in gastric mucosa of patients and *H. pylori* infected mice with strains encoding CagA. *In vitro* CagA induces IL-33 production by gastric epithelial cells, leading to ILC2 activation and IL-5 production (10, 11). Similarly, increased *IL7* mRNA levels are found in the gastric mucosa of *H. pylori* infected patients (64). In the stomach, ILC2 accumulation and activation is dependent on IL-7 and IL-33, respectively (63). ILC2s coordinate with B cells to produce *H. pylori*-specific IgA which can coat bacteria in the stomach in a mouse model (63).

Non-gastric *Helicobacter* species, such as *H. apodemus* and *H. typhlonius*, have different effects on ILCs compared to *H. pylori* (12). In mice lacking T and B cells, colonization of the GI tract with these two *Helicobacter* species causes activation of ILCs with increased frequencies of IL-22- and/or IL-17-producing ILC3s and IFNγ-producing ILC1s, resulting in GI inflammation. However, infection reduces ILC3 number, particularly T-bet-expressing ILC3s (12). How these *Helicobacter* species cause reduction in ILC3 numbers remains unknown. The reduction in ILC3s was only observed in mice lacking adaptive immune cells and not wild-type mice suggesting that adaptive immunity can sustain ILC3s during *Helicobacter* colonization.

### Campylobacter

*Campylobacter* species, mainly *C. jejuni* and *C. coli*, are the causative agents of one type of food-borne gastroenteritis (65). Although these Gram-negative infections are mostly self-limiting, long-term immune related intestinal dysfunction has been associated with *Campylobacter* infections (66, 67). Several studies have examined the role of ILCs during *Campylobacter* infection, using a mouse model with a predisposition to intestinal inflammation due to a lack of IL-10 signaling (13, 68, 69). IL-23, a primary activator of ILC3s, is key driver of inflammation during *Campylobacter* infection (69). Jing et al. found that IL-23 regulated production of IFNγ, IL-17 and IL-22 by ILC1s and ILC3s during *Campylobacter* infection (69). Consistent with previous studies, IFN-γ and IL-17 promoted inflammation while IL-22 was found to be dispensable for inflammation during *Campylobacter* infection (69). This contrasts with another study in which IL-22 was protective in a mouse model of *Campylobacter* where IL-10 signaling was intact (70).

*Campylobacter* infection induces production of IFNγ, IL-17 and IL-22 from both ILCs and T cell subsets in mice (68). Similar observations were reported using an *ex vivo* human gut model of infection (71). Both IFNγ and IL-17 were found to promote intestinal inflammation during *Campylobacter* infection (68). Two recent studies found that ILC-derived IFNγ contributes to intestinal pathology during infection (13, 69). While ILC1s represent a major source of IFNγ, ILC3s can also convert into IFN-γ-producing ILC1s by expressing the transcription factor T-bet (5, 41). Interestingly, *Campylobacter* infection induces conversion of ILC3s to ILC1s and these ex-ILC3s in turn produce IFNγ that promotes intestinal inflammation (13). IL-12 and IL-23 have been shown to facilitate *in vitro* ILC3-ILC1 plasticity (72) and both cytokines are elevated during *Campylobacter* infection (13, 68). One may speculate that dysregulation of IL-12 and IL-23 levels during *Campylobacter* infection promotes ILC3 to ILC1 conversion. However, the mechanism by which *Campylobacter* regulates ILC3 to ILC1 conversion has not yet been elucidated. *Campylobacter* spp. produce many toxins that modulate host cells, with the best studied being cytolethal distending toxin (CDT) from *C. jejuni*, that may modulate ILC function. Together, these studies show that ILCs are a major player in inducing intestinal inflammation and promote pathology during *Campylobacter* infection.

### Bacillus anthracis

*B. anthracis* is the causative agent of anthrax, a rare but deadly infection of the lungs, GI tract or skin (73). The major virulence factors of the Gram-positive *B. anthracis* are lethal toxin (LT) and edema toxin (ET) (73). LT or ET can both suppress immune function of both innate and adaptive immune cells (74–76). In line with the immunosuppressive action of anthrax toxins, LT can suppress ILC3 activation both *in vitro* and *in vivo* in a mouse model (14). LT, a zinc metalloprotease, cleaves mitogen activated protein kinases perturbing cell signaling (77). LT reduced IL-22 production by IL-23-activated ILC3s by inactivating MAPK signaling (14). In contrast, ET as an adenyl cyclase, elevates intracellular cAMP levels in ILC3s leading to their *in vitro* activation (15). How LT, ET and ILC3s interact *in vivo* remains to be determined. It is likely that LT-mediated suppression may overcome ET-mediated activation of ILC3s as a study found suppression of the mRNA encoding IL-22-induced antimicrobial peptides, REG3β and REG3γ, in the colons of *B. anthracis* infected mice (75). ET effects can be dose-dependent, and the toxin can suppress T cell proliferation or promote Th17 differentiation (76, 78), which may have similar implications on related ILC3s. Thus, it is also possible that ET may exert suppressive action on ILC3s *in vivo*. Although not a focus of our review and has yet to be studied, these toxins likely modulate ILCs in the lungs during respiratory disease. Overall, *B. anthracis* and its two major toxins modulate ILC3 function.

## Crosstalk between ILCs and the adaptive immune system

Although ILCs are mainly tissue-resident cells residing at mucosal surfaces, they are also found in the peripheral blood, bone marrow, and primary and secondary lymphoid organs (79–81). Recently, migratory ILCs have been reported in context of infection (42). Their location in lymphoid organs where the adaptive immune response is initiated suggests a role for ILCs in shaping adaptive immunity. Indeed, several studies have underscored the role of ILCs in regulation of adaptive immunity (reviewed in (81, 82)). ILCs can directly or indirectly influence both T and B cell-mediated responses (82, 83). As one of the first responders to pathogens, ILCs influence the cytokine milieu and thus also influence adaptive immune response (82, 83). ILC2s and ILC3s can also directly interact with T cells by expressing MHCII and acting as antigen presenting cells (APCs) (84–86). ILCs can also express co-stimulatory or auxiliary molecules such as OX40L, ICOS/ICOSL, and PD-L1 (the latter two reported only for ILC2s) which facilitates a direct interaction with T cells thus modulating the adaptive immune response (87–91). Conversely, the adaptive immune system can also regulate ILC function. For example, T cell-derived IFNγ directly limits ILC2 function (92, 93) and regulatory T cells can control ILC2 and ILC3 responses (94–96). T cells can also enhance ILC2 responses via direct interaction with ILC2s mediated by MHCII or other auxiliary molecules listed above (86, 97). Thus, crosstalk between ILCs and adaptive immune cells shape adaptive immunity as well ILC function.

In the healthy GI tract, ILC3s regulate the immune response to the microbiota by regulating T and B cell responses. ILC3s acting as APCs interact with T cells resulting in clonal deletion of microbiota-specific T cells (85). Similarly, ILC3s can act as APCs to interact with T helper follicular cells to regulate the immunoglobulin A (IgA) response to the microbiota (98). ILC3s can also indirectly support IgA production via lymphotoxin signaling in the intestine (99). Ablation of lymphotoxin α signaling in ILC3s results in inhibition of IgA production and composition changes of the GI microbiota (99). Studies on ILC crosstalk during enteric bacterial infection are very limited. As discussed above, ILC3s are important in defense against enteric pathogens such as *C. rodentium* (21). Several studies have dissected the spatiotemporal interplay of ILCs and the adaptive immune response during *C. rodentium* infection with ILCs being critical during the early stage of infection and the adaptive response acting later to clear infection (100–102). IL-22, the signature cytokine of ILC3s, regulates the organization and maintenance of colonic lymphoid structures during *C. rodentium* infection by acting downstream of lymphotoxin signaling (103). Colonic lymphoid structures contain T and B cells and thus are sites of initiation of the adaptive immune response. A recent study showed that *Salmonella* infection induced migration of intestinal RORγt^+^ T-bet^+^ ILCs to mesenteric lymph nodes which contributed to the protective IFNγ response (42). Whether these migratory ILCs contribute to the overall adaptive immune response remains unknown. During colonization by non-gastric *Helicobacter* species, the adaptive immune system sustains ILC3 number (12). With progress in our understanding of ILC biology, the interplay of ILCs and adaptive immune system has become apparent during healthy, steady-state conditions. However, more studies are needed to uncover the specific nature of ILC and adaptive immune system interactions during enteric infections which will facilitate development of better therapeutics.

## Targeting ILCs for treatment of enteric bacterial infections

The standard of care for bacterial GI diseases is antibiotics. However, with the ever-growing rise of antibiotic resistance, this treatment may fail for some patients. Targeting the host immune response, or in combination therapy with antibiotics, has the potential to become an effective treatment to combat GI infections. Although ILCs are rare immune cells, they are critical regulators of tissue homeostasis, inflammation, and immunity against GI infections (5). Increased understanding of ILC biology in recent years has resulted in development of new or co-opting T cell-targeted therapies to therapeutically target ILCs as well (104). Existing ILC targeting strategies for therapeutics include administration of cytokines, adoptive transfer of ILCs, antibodies against ILC-related cytokines, ILC depletion, modulation of ILC plasticity, migration and function, and microbiota manipulation (104). These take advantage of pathways that were developed based on T cell biology. Development of treatments that precisely target ILCs and not T cells is a challenge for the field. Furthermore, manipulation of oxygen levels in the GI tract may be an effective means to counteract a pathogen. Thus, targeting ILCs is increasingly considered for clinical therapy as evident by several preclinical or clinical studies on ILC-targeting therapies (104, 105). In fact, several biological therapies targeting ILCs are already approved, and multiple others are in development for Crohn’s disease, a chronic inflammatory disease of GI tract (104, 106).

As discussed, enteric bacterial pathogens often modulate ILC function contributing to a pathologic or defensive outcome. Pathogen-induced cytokine production by ILCs can be to the pathogen’s advantage or contribute to protection of the host. Further, ILC plasticity and migration can also be modulated by enteric bacterial pathogens. Hence, therapeutics targeting ILC biology are an attractive novel option for the treatment of GI infections. Existing ILC-targeting biologic therapeutics could be adapted to treat enteric bacterial infections. Although studies have started to highlight the roles of ILCs in the context of different enteric bacterial infections, more studies on defining the precise roles of ILCs during infection, especially in humans, are needed before existing ILC-targeting strategies can be adapted to treating enteric infections. Such studies will provide a comprehensive understanding of ILC biology in context of infection leading to adaptation of existing or development of new therapeutic strategies targeting ILCs for enteric bacterial infections.

## Conclusions

Despite their relatively recent discovery, ILCs have emerged as key players in barrier resistance to prevent breach by pathogens. ILCs are among the first immune cells that these pathogens encounter. ILCs during enteric bacterial infections can have both protective and pathogenic roles depending on the pathogen and context of infection. Enteric pathogens have evolved strategies to evade host immune responses to help establish their infection in the GI tract. In only a few interactions have we identified the specific bacterial virulence or effector molecules that manipulate specific signaling pathways in ILC3s. Future studies need to examine these different pathogens in the shared and novel ways they can modulate ILC responses. Further, many of these findings are established from animal infection models. There is a need for validation in context of human infection to increase the translation of these basic research studies to better patient outcomes. A better understanding of ILC biology in the context of infection has high potential to lead to ILC-targeted therapies for enteric bacterial infections.

## Author contributions

PS contributed to the conceptualization and wrote the first draft. LZ contributed to the conceptualization, reviewed and edited the article, and generated the figure. All authors contributed to the article and approved the submitted version.
